# Acetylation-dependent glutamate receptor GluR signalosome formation for STAT3 activation in both transcriptional and metabolism regulation

**DOI:** 10.1038/s41420-020-00389-6

**Published:** 2021-01-14

**Authors:** Xiang-Rong Li, Xiaju Cheng, Jia Sun, Yan S. Xu, Nannan Chen, Yimei Hao, Chao Huang, Y. Eugene Chin

**Affiliations:** 1 Jiangsu Key Laboratory of Infection and Immunity, Institutes of Biology and Medical Sciences, Soochow University, 215123 Suzhou, Jiangsu China; 2 Cancer Research Center, Shandong University School of Medicine, 250012 Jinan, Shandong China; 3 Key Laboratory of Tissue Microenvironment and Tumor, CAS Center for Excellence in Molecular Cell Science, Shanghai Institute of Nutrition and Health, University of Chinese Academy of Sciences, Chinese Academy of Sciences, Shanghai, China; 4 School of Life Sciences, Center for Life Sciences, Yunnan University, 650091 Kunming, China

**Keywords:** Acetylation, CNS cancer

## Abstract

Besides their original regulating roles in the brain, spinal cord, retina, and peripheral nervous system for mediating fast excitatory synaptic transmission, glutamate receptors consisting of metabotropic glutamate receptors (GluRs) and ionotropic glutamate receptors (iGluRs) have emerged to have a critical role in the biology of cancer initiation, progression, and metastasis. However, the precise mechanism underpinning the signal transduction mediated by ligand-bound GluRs is not clearly elucidated. Here, we show that iGluRs, GluR1 and GluR2, are acetylated by acetyltransferase CREB-binding protein upon glutamate stimulation of cells, and are targeted by lysyl oxidase-like 2 for deacetylation. Acetylated GluR1/2 recruit β-arrestin1/2 and signal transducer and activator of transcription 3 (STAT3) to form a protein complex. Both β-arrestin1/2 and STAT3 are subsequently acetylated and activated. Simultaneously, activated STAT3 acetylated at lysine 685 translocates to mitochondria to upregulate energy metabolism-related gene transcription. Our results reveal that acetylation-dependent formation of GluR1/2–β-arrestin1/2–STAT3 signalosome is critical for glutamate-induced cell proliferation.

## Introduction

Glutamate receptors (GluRs), the major excitatory receptor in the brain, are characterized as ionotropic or metabotropic. Ionotropic GluRs are tetrameric ligand-gated cation channels that induce depolarization of the postsynaptic membrane, following the presynaptic release of glutamate. Their actions underlie the cellular models of learning and memory, modulate the excitability of neuronal networks, and are required for synaptic maturation. Ionotropic GluRs can be pharmacologically classified according to their sensitivity to AMPA, Kainate, and NMDA. AMPA receptors (GluR1–4) evoke excitatory postsynaptic potentials and mediate fast synaptic transmission. In contrast, Kainate receptors (GluR5 and 6 and KA1/2) and NMDA receptors (NR1–3) mediate slower synaptic transmission exert effects on plasticity.

In addition to the well-established role of the glutamatergic system in the central nervous system (CNS), evidence is emerging of a role for glutamate and its receptors in peripheral tissues^1^ and in cancer^2,3^. It has been demonstrated that GluR subunits are expressed in a variety of cancer cell lines and tumors, i.e., glioma, colorectal and gastric cancer, oral squamous cell carcinoma, prostate cancer, et al.^4–8^. However, precise mechanism underlying the functional role of GluRs in cancer initiation and progression is unclear.

Acetylation is one of the major posttranslational protein modifications in the cell, with manifold effects on the protein level, as well as on the metabolome level^9^. Beside essential role of acetylation in function of histone, acetylation is implicated in regulating numerous nonhistone transcription-regulating proteins, including transcription factors, transcriptional co-activators, cytokine/growth factor receptors, and nuclear receptors^10^. Thus, regulation of cell signaling transduction and gene transcription are major roles of nonhistone protein acetylation.

Signal transducer and activator of transcription 3 (STAT3) in the cytoplasm is activated by cytokines or growth factors present in the cellular environment^11^. STAT3 proteins activated by cytokines or growth factors undergo posttranslational modifications, including tyrosine and serine phosphorylation, acetylation, and methylation^12–15^. STAT3 shuttles between the cytoplasm and nucleus in response to phosphorylation, and DNA binding and promoter initiation by nuclear STAT3 is terminated via dephosphorylation^11,16^. Shuttling between the cytoplasm and mitochondria is regulated by reversible acetylation at K685 in STAT3 (ref. ^17^). STAT3 has been shown to be activated by glutamate through both ionotropic and metabotropic glutamate receptors (mGluRs), but mechanism underlying STAT3 activation by glutamate is not elucidated^18,19^.

Arrestins were first discovered as a part of a conserved two-step mechanism for regulating the activity of G protein-coupled receptors (GPCRs) in the visual rhodopsin system and in the β-adrenergic system^20–22^. In response to a stimulus, GPCRs activate heterotrimeric G proteins. In order to turn off this response, or adapt to a persistent stimulus, active receptors need to be desensitized. The first step is phosphorylation by a class of serine/threonine kinases called G protein-coupled receptor kinases (GRKs). GRK phosphorylation specifically prepares the activated receptor for arrestin binding. Arrestin binding to the receptor blocks further G protein-mediated signaling and targets receptors for internalization, and redirects signaling to alternative G protein-independent pathways, such as β-arrestin signaling. In addition to GPCRs, arrestins bind to other classes of cell surface receptors and a variety of other signaling proteins^8^.

Here, we show that ionotropic glutamate receptors (iGluRs), GluR1/2, are acetylated by CREB-binding protein (CBP) upon glutamate stimulation. Acetylation of GluR1/2 recruit β-arrestin1/2 and STAT3 to form a signalosome, followed by acetylation of STAT3 to translocate to mitochondria, and simultaneously, activation of mTOR and extracellular signal-regulated kinase 1/2 (ERK1/2) signaling pathway to increase protein synthesis and cell proliferation. Our results reveal a novel acetylation-dependent mechanism underlying glutamate-induced cell growth.

## Results

### Glutamate-induced acetylation of both GluR1 and GluR2

**Fig. 1:**
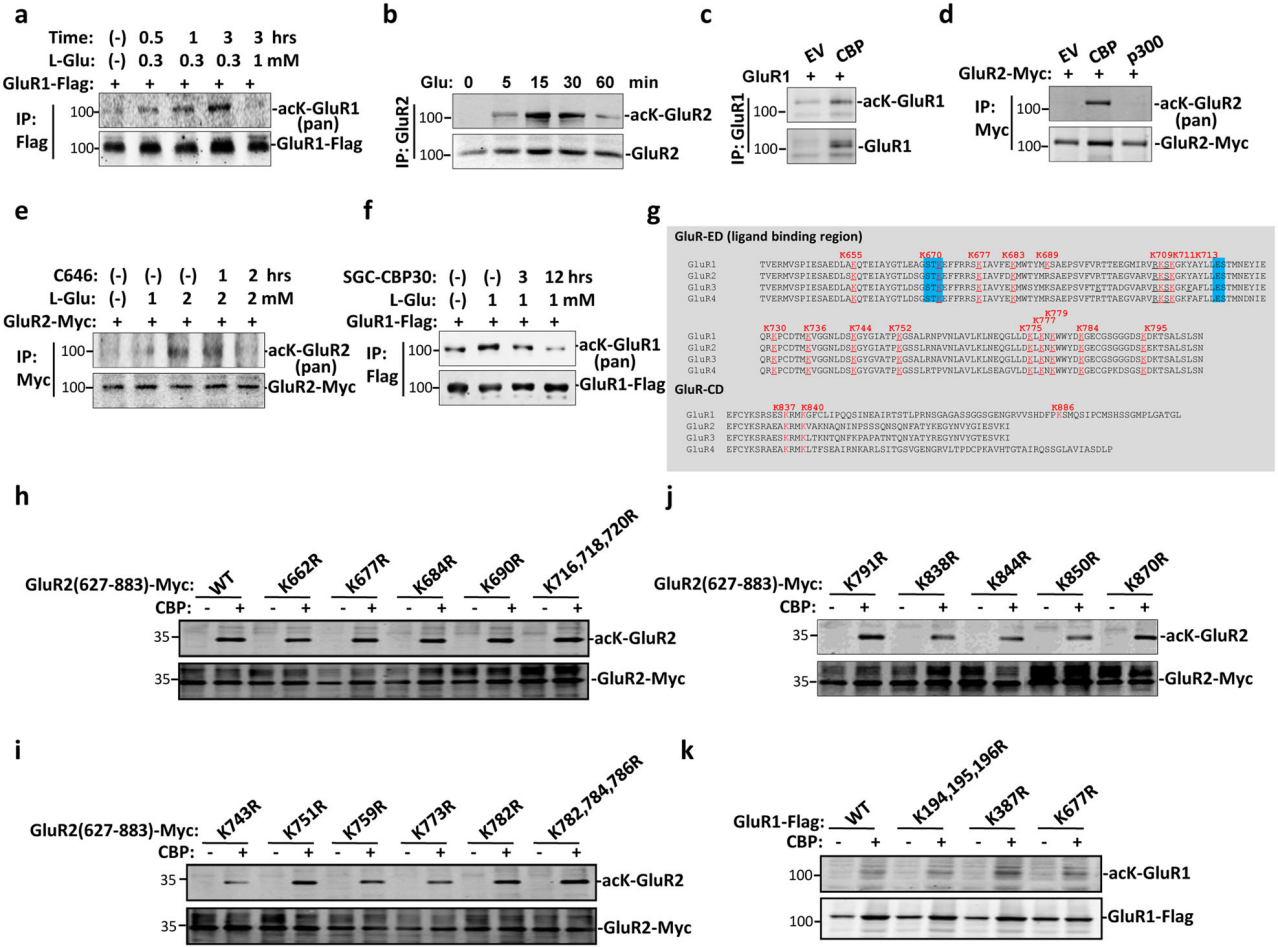
GluR1 and 2 are acetylated by CBP upon glutamate treatment. **a** 293 T cells were transfected with Flag-GluR1 and then were treated with glutamate for different time. Cells were subjected to immunoprecipitation with Flag antibody followed by western blot with pan-acetyl and Flag antibodies. Data shown are representative of three independent experiments. **b** C6 cells were treated with glutamate for different time. Cells were subjected to immunoprecipitation with GluR2 antibody followed by western blot with pan-acetyl and GluR2 antibodies. **c** 293 T cells were either transfected with GluR1 and CBP or GluR1 alone. Cells were subjected to immunoprecipitation with GluR1 antibody followed by western blot with pan-acetyl and GluR1 antibodies. **d** 293 T cells were transfected with indicated plasmids. Cells were subjected to immunoprecipitation with Myc antibody followed by western blot with pan-acetyl and Myc antibodies. **e** 293 T cells were transfected with Myc-GluR2 and then were treated with glutamate and CBP inhibitor (C646) for different time. Cells were subjected to immunoprecipitation with Myc antibody followed by western blot with pan-acetyl and Myc antibodies. Data shown are representative of two independent experiments. Data shown are representative of two independent experiments. **f** Alignment of C-terminal region of GluRs family. **g** Schematic show of GluR1 structure and possible sites of acetylation. **h**–**k** Mapping of acetylation sites in GluR1 and GluR2 by mutation analysis.

To explore the role of acetylation in iGluR, GluR1 and GluR2, signaling transduction, we first checked if GluR1 and 2 are targeted by acetylation. C6 cells were treated with glutamate for different times and GluR1/2 acetylation status was tested. While GluR1 acetylation reached its peak after 30 min treatment with glutamate, acetylation peak of GluR2 occurred after treatment with glutamate for only 15 min (Fig. 1a, b). Acetylation level of both GluR1 and 2 gradually reduced post glutamate treatment of cells for over 60 min (Fig. 1a, b). Next, we transfected cells with either Flag-GluR1 or Myc-GluR2 with or without acetyltransferase CBP or p300, and then immunoprecipitation and western blot was performed to test GluR1/2 acetylation status. Results show that both GluR1 and GluR2 were subjected to acetylation by CBP (Fig. 1c, d). Furthermore, glutamate-stimulated acetylation of GluR1 and GluR2 were reduced after inhibition of CBP by CBP inhibitor SGC-CBP30 or C646, respectively (Fig. 1e, f). It was confirmed that CBP mediate GluR1 and GluR2 acetylation stimulated by glutamate. GluRs family members (GluR1–4) contain several conserved lysine in both extracellular ligand-binding region and intracellular protein segments (Fig. 1g). To identify acetylation site, several conserved lysine in GluR1 and GluR2 were mutated to arginine. However, we failed to identify accurate acetylation site, probably due to global acetylation of GluR1 and GluR2 induced by overexpression of CBP (Fig. 1h–k). Altogether these results show that GluR1 and 2 were acetylated by CBP upon glutamate treatment.

### Acetylated GluR2 was targeted by Loxl2 for deacetylation

**Fig. 2:**
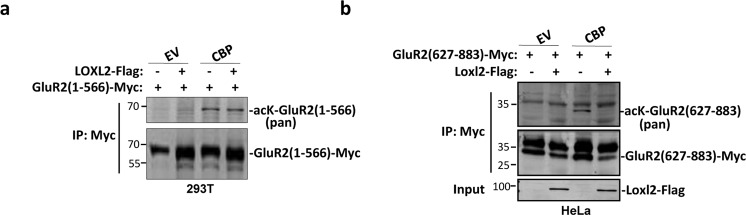
GluR2 is deacetylated by Loxl2. **a**, **b** 293 T cells were transfected with indicated plasmids. Cells were subjected to immunoprecipitation with Myc antibody followed by western blot with pan-acetyl and Myc antibodies.

We and others have shown that Lysyl oxidase-like 2 and 3 (Loxl2/3) also function as demethylase or deacetylases^23,24^, we therefore tested whether Loxl2 can deacetylate GluR2. Although GluR2 N-terminal part was acetylated by CBP, it was not targeted by Loxl2 for deacetylation (Fig. 2a). On the contrary, GluR2 C-terminal part was efficiently deacetylated by Loxl2 (Fig. 2b). These results indicated that GluR2 C-terminal part is subjected to acetylation regulation by CBP and Loxl2.

### Glutamate-induced acetylation-dependent GluR1/2–β-arrestin1/2–STAT3 complex formation

**Fig. 3:**
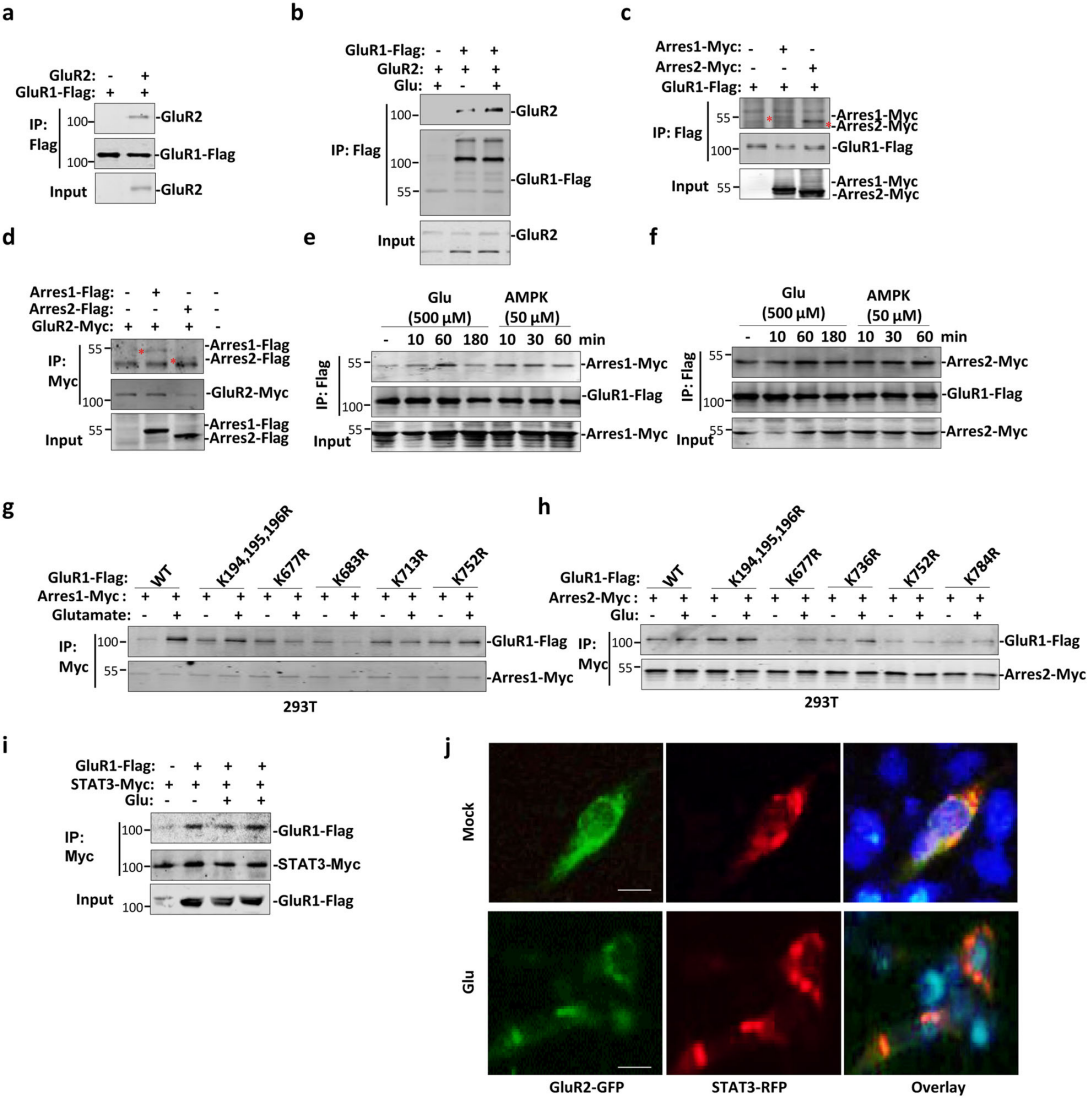
GluR1/2-β–arrestin1/2–STAT3 complex formation is dependent on GluR1 and GluR2 acetylation. **a** 293 T cells were transfected with GluR1 or GluR1 plus GluR2. Cells were subjected to immunoprecipitation with Flag antibody followed by western blot with GluR2 and Flag antibodies. **b** 293 T cells were transfected with indicated plasmids followed with or without treatment of glutamate. Cells were subjected to immunoprecipitation with Flag antibody followed by western blot with GluR2 and Flag antibodies. **c**, **d** 293 T cells were transfected with indicated plasmids. Cells were subjected to immunoprecipitation with Flag antibody followed by western blot with Myc and Flag antibodies. **e**, **f** Cells were treated with either glutamate or AMPK for different time, cells were subjected to immunoprecipitation with Flag antibody followed by western blot with Myc and Flag antibodies. Data shown are representative of three independent experiments. **g**, **h** Mutation assay of acetylation sites that affected interaction between GluR1 and arrestin1/2. 293 T cells were transfected with indicated plasmids and then were treated with or without glutamate. Cells were subjected to immunoprecipitation with Myc antibody followed by western blot with Myc and Flag antibodies. **i** 293 T cells were transfected with indicated plasmids and then were treated with or without glutamate. Cells were subjected to immunoprecipitation with STAT3 antibody followed by western blot with Myc and Flag antibodies. Data shown are representative of two independent experiments. **j** 293 T cells were transfected with GluR2-GFP and Stat-RFP. Localization of GluR2 and STAT3 was observed by microscopy. Scale bar: 10 μm. Data shown are representative of three independent experiments.

GluRs family members usually form heterotetramer (dimer of dimers) to carry out ion channel function, therefore, we tested whether this dimerization is an acetylation-dependent or -independent process. As reported before^25^, GluR1 forms a complex with GluR2 (Fig. 3a). Interestingly, interaction between GluR1 and 2 was enhanced after treatment of cells with glutamate (Fig. 3b). As mGluRs bind with β-arrestin1/2, which regulate mGluR downstream signaling^26,27^, we therefore would like to know if GluR1/2 also interact with β-arrestin1/2. Cells were transfected with β-arrestin1/2 with either GluR1 or 2, and immunoprecipitation was performed to show that GluR1 or 2 interacted with both β-arrestin1 and 2 (Fig. 3c, d). Furthermore, both agonist glutamate and AMPA enhanced binding of β-arrestin1/2 to GluR1 (Fig. 3e, f). Interestingly, while glutamate-induced binding of GluR1 to β-arrestin1 was greatly impaired by mutation of lysine 677 and 683 in extracellular region of GluR1, interaction between GluR1 and β-arrestin2 was impaired by mutation of lysine 677, 752, and 784 to arginine (Fig. 3g, h). β-arrestin1 has been shown to bind to STAT3 upon IL-6 stimulation^28^, we therefore tested if GluR1 and 2 also interact with STAT3 and therefore, forms a protein complex containing GluR1/2, β-arrestin1/2, and STAT3. Immunoprecipitation and fluorescence microscopy showed that both GluR1 and GluR2 constitutively interacted with STAT3 with or without addition of glutamate, respectively (Fig. 3i, j). Altogether, these results suggest that GluR1/2, β-arrestin1/2, and STAT3 form a complex to function in GluR signaling transduction.

### STAT3 acetylation and translocation to mitochondria is stimulated by glutamate

**Fig. 4:**
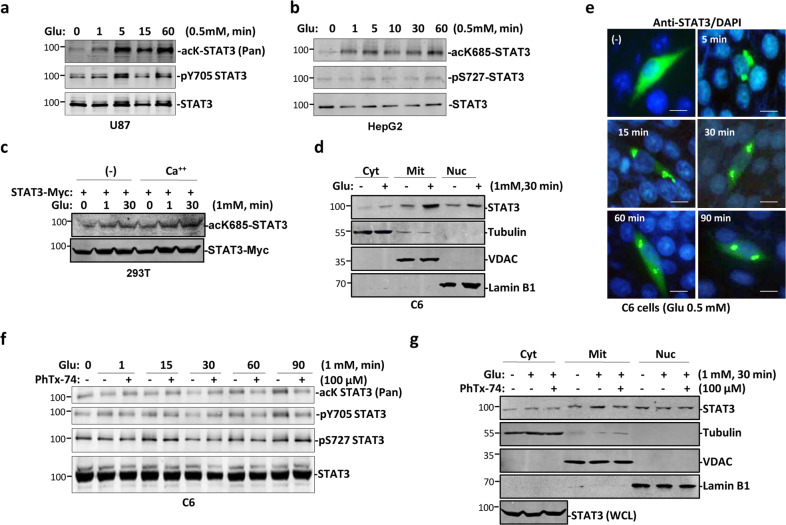
Glutamate-stimulated translocation of STAT3 into mitochondria. **a**, **b** U87 **a** and HepG2 **b** cells were treated with glutamate, followed by western blot with indicated antibodies. Data shown are representative of two independent experiments. **c** 293 T cells were transfected with STAT3 and treated with either calcium or glutamate, or both. Cells were lysed and blotted with indicated antibodies. Data shown are representative of three independent experiments. **d** C6 cells were treated with or without glutamate and followed by fractionation of cell. Cells were lysed and blotted with indicated antibodies. **e** C6 cells were treated with glutamate for different time, and then cells were immunostained with anti-STAT3 antibody. Cells were countstained with DAPI. Scale bar: 10 μm. Data shown are representative of two independent experiments. **f** C6 cells were treated with different combination of glutamate and PhTX-74. Cells were lysed and blotted with indicated antibodies. **g** C6 cells were treated with different combination of glutamate and PhTX-74. Cells were then lysed and fractionated, and blotted with indicated antibodies.

As interaction between GluR1/2 and β-arrestin1/2 was stimulated by glutamate, and STAT3 was constitutively bound with GluR1/2, it is possible that STAT3 is activated upon this complex formation. We therefore checked activation status of STAT3. Levels of both phosphorylation at Y705 and acetylation at K685 in STAT3 were elevated upon glutamate treatment for a period of time (Fig. 4a, b). STAT3 phosphorylation at S727 was not changed after addition of glutamate (Fig. 4b). GluR1/2 can form calcium channel upon glutamate stimulation, we therefore tested whether calcium flow would also activate STAT3. Both glutamate and calcium enhanced acetylation level at K685 in STAT3 (Fig. 4c). At the same time, translocation of STAT3 into mitochondria was enhanced by glutamate (Fig. 4d, e), suggesting a manner of acetylation-dependent mitochondria translocation of STAT3, as reported previously^17^. To confirm the role of glutamate-induced STAT3 acetylation and translocation into mitochondria, cells were treated with philanthotoxin-7,4 (PhTx-74), a potent and selective antagonist of iGluRs^29^. Longer time incubation of cells with PhTx-74 reduced acetylation level of STAT3 and subsequent STAT3 translocation into mitochondria simultaneously (Fig. 4f, g). All these results showed that Stat3 was acetylated and activated by glutamate for translocation into mitochondria.

### Glutamate induces accelerated cell proliferation

**Fig. 5:**
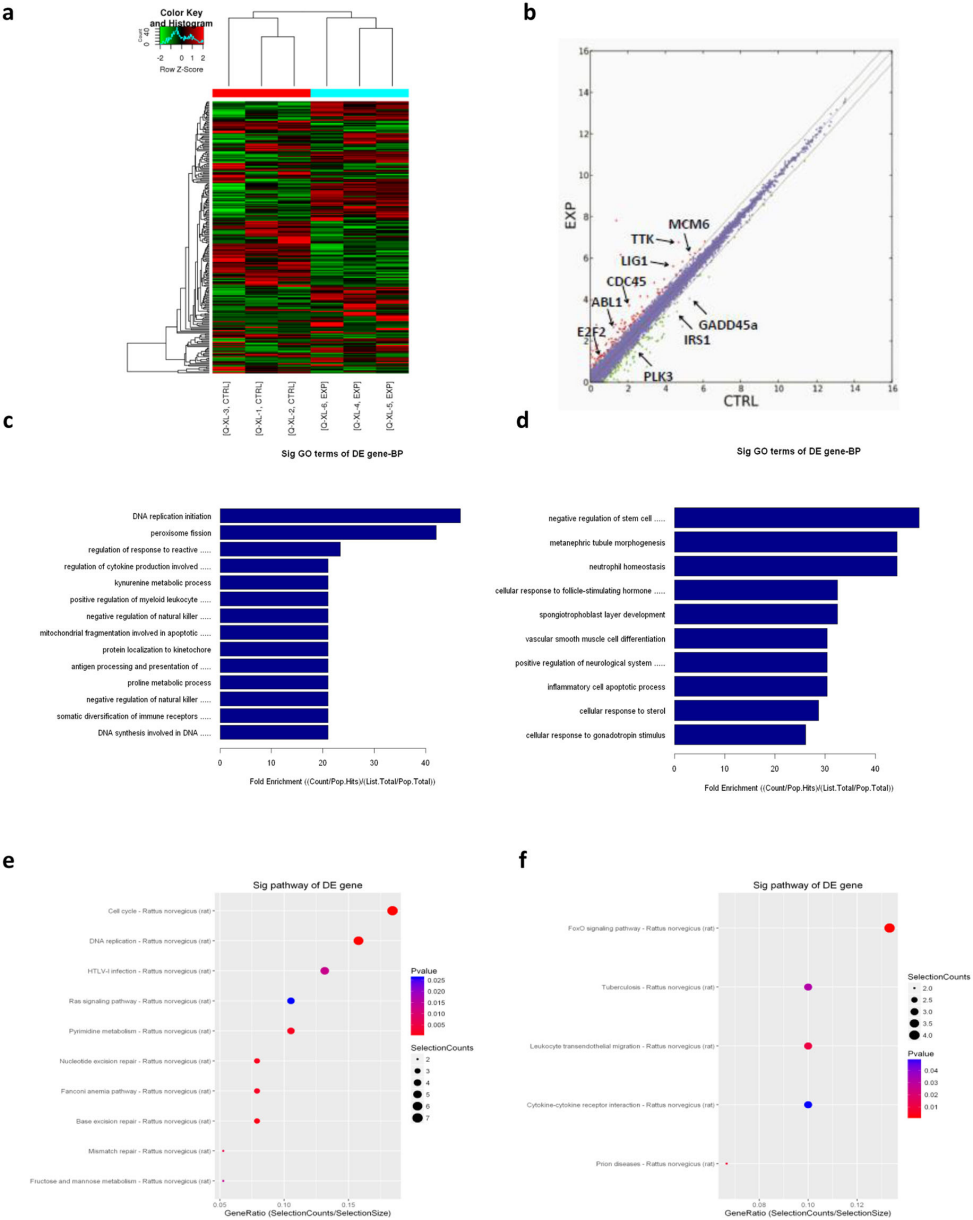
Microarray analysis of glutamate-treated cells. **a** Raw data of microarray. **b** Analysis of differentially expressed genes in glutamate-treated cells compared to mock-treated cells. **c**, **d** Upregulated (**c**) or downregulated (**d**) biological processes in glutamate-treated cells compared to mock-treated cells. **e**, **f** Upregulated (**e**) or downregulated (**f**) signaling pathways in glutamate-treated cells compared to mock-treated cells.

To further investigate biological function of glutamate stimulation of cells, we performed RNA-seq before and after addition of glutamate into cultured C6 cells (Fig. 5a). Scatter plot showed that expression of genes related to DNA replication and cell cycle regulation, such as POLE, CDC45, MCM6, and TTK, were upregulated, whereas expression of genes that have been shown to be negative regulators of mitotic cell cycle, such as PLK3, GADD45a, and IRS1, were downregulated upon glutamate treatment, suggest increased entry into S phase and enhanced cell cycle progression of C6 cells after addition of glutamate (Fig. 5b). Gene ontology analysis of biological process further revealed that DNA replication initiation was enhanced (Fig. 5c), however, we also observed enhanced cell differentiation (Fig. 5d). Analysis of KEGG signaling pathway further revealed accelerated cell cycle progression and reduced Foxo-related signaling transduction, which is a negative regulator of G1/S transition of the cell cycle^30^ (Fig. 5e, f).

### GluR1/2–β-arrestin1/2–STAT3 signalosome is required for glutamate-induced cell proliferation

**Fig. 6:**
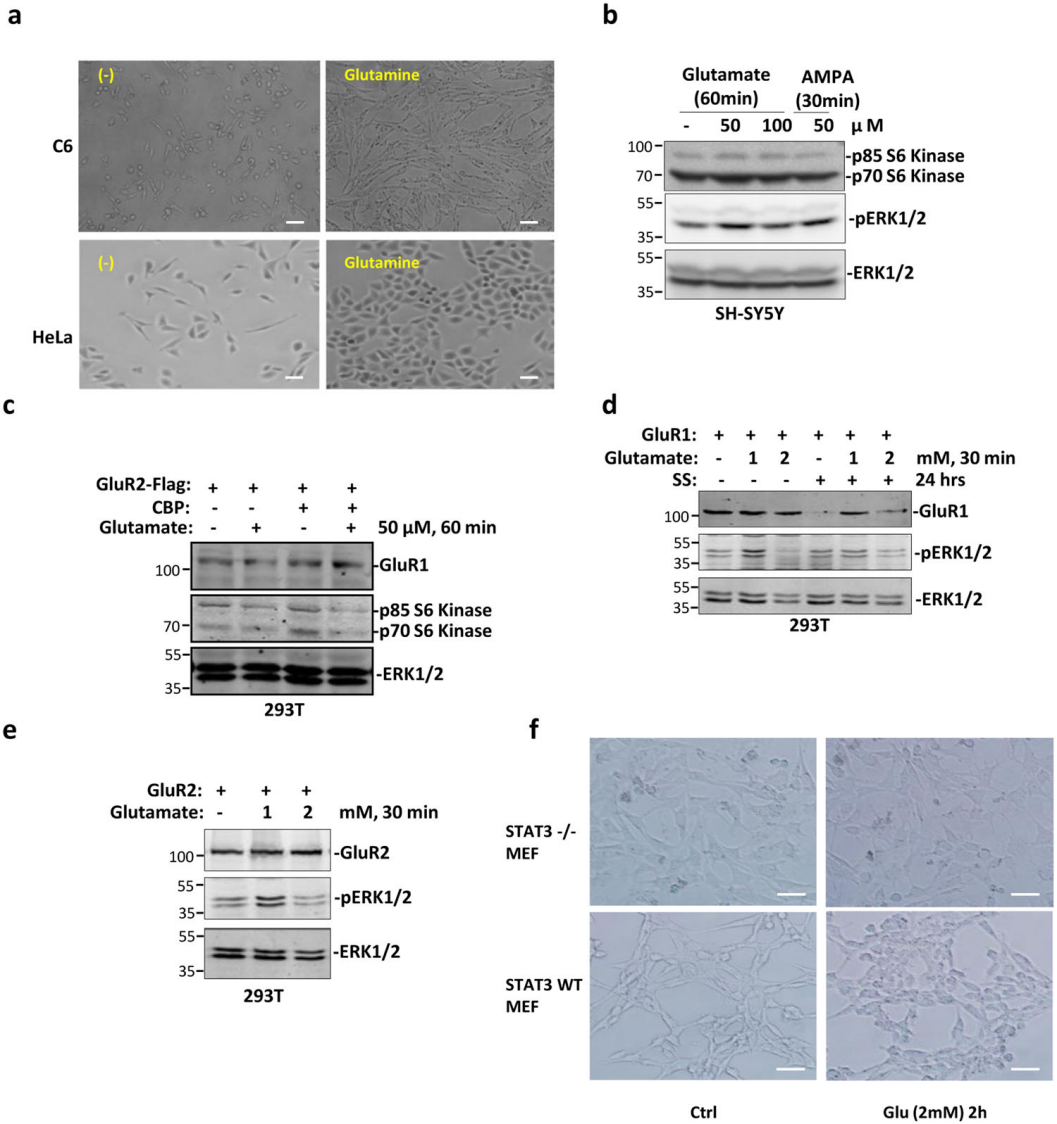
Glutamate-induced cell proliferation is mediated by STAT3. **a** C6 and Hela cells were treated with glutamine and cell proliferation was observed by microscopy. Scale bar: 50 μm. Data shown are representative of three independent experiments. **b** SH-SY5Y cells were with either glutamate or AMPK, cells were lysed and blotted with indicated antibodies. Data shown are representative of two independent experiments. **c** 293 T cells were transfected with indicated plasmids and then were treated with or without glutamate. Cells were lysed and blotted with indicated antibodies. **d** 293 T cells were transfected with GluR1 followed by treatment with glutamate or serum starvation. Cells were lysed and blotted with indicated antibodies. **e** 293 T cells were transfected with GluR2 followed by treatment with glutamate for different time. Cells were lysed and blotted with indicated antibodies. **f** Wild-type and STAT3^−/−^ MEF cells were treated with glutamate. Cell proliferation was observed by microscopy. Scale bar: 50 μm. Data shown are representative of two independent experiments.

Except in CNS, GluRs are also widely expressed in various cancer cells^31^. To further assess the role of formation of GluR1/2–β-arrestin1/2–STAT3 signalosome in cancer cell growth, cells were treated with glutamine and accelerated cell growth was observed (Fig. 6a). We then checked activation of mTOR pathway, which is required for rapid protein synthesis and cell proliferation^32^. Both glutamate and AMPA treatment of cells induced upregulation of protein level of both p70 and p85 S6 kinases, direct targets of activated mTOR kinase^32^ (Fig. 6b). Moreover, ERK1/2, which is required for cell proliferation^33^, was activated by glutamate, but not by AMPA (Fig. 6b). Upregulation of acetylation level by overexpression of CBP also increased p70/p85 S6 kinase expression (Fig. 6c), suggesting the role of acetylation in glutamate-induced mTOR activation. To support observations mentioned above, ERK1/2 was activated by treatment with low concentration of glutamate in cells overexpressing GluR1 or GlulR2 (Fig. 6d, e). To test the role of STAT3 in glutamate-induced cell proliferation, wild-type and STAT3 knock out (STAT3^−/−^) MEF cells were treated with glutamate and phase-contrast microscopy showed that deletion of STAT3 greatly impaired cell growth induced by glutamate (Fig. 6f). Together, these results indicated that glutamate-induced cell proliferation is partially mediated by activation of mTOR and ERK1/2 signaling pathways.

## Discussion

GluR1 and GluR2 are four-transmembrane proteins that belong to the glutamate-gated ion channel family. GluR1 only interacts with GluR2 to form a functional heterotetrameric GluR^25^. In addition to constitutive phosphorylation on a key serine 831 in the C-terminal extracellular region of GluR1, neurotransmitter-induced phosphorylation of another key serine by CaMKII leads to a potentiation of glutamate-mediated current^34^. GluR2 is the most widely expressed of four AMPA receptor subunits and is regulated both by palmitoylation and phosphorylation. Palmitoylation at C610 in GluR2 results in decreased surface expression. Phosphorylation by PKC on S880, and by Src on Y876 in GluR2, induces its internalization^25^. All these research indicating importance of posttranslational modification in regulating GluR1/2 physiological function.

Although acetylation of nonhistone proteins, including cytokine receptors is involved in key cellular processes relevant to physiology and disease, such as gene transcription, DNA damage repair, cell division, signal transduction, protein folding, autophagy, and metabolism, their exact functions in signaling transduction are not completely elucidated^10,35,36^. Acetylation has been shown to maintain GluR1 stability and reduce receptor trafficking in rat neuron, and elevated acetylation of GluR1 by knockdown of deacetylases Sirt2 leads to impaired synaptic plasticity and memory^37^. As GluR1/2 are ubiquitously expressed in nonneuronal cell, including tumor cells^31,38^, questions emerged as whether GluR1/2 in those cells are also subjected to acetylation and, if so, what the significances of GluR1/2 acetylation?.

We showed here that GluR1/2 are acetylated by acetyltransferase CBP upon glutamate stimulation of various tumor cells, and acetylated GluR1/2 can be deacetylated by Loxl2. Consistent with enhanced protein stability by acetylation in neuron^37^, we observed increased GluR1/2 protein level by CBP cotransfection or glutamate stimulation in tumor cells, suggesting general role of acetylation in maintaining GluR1/2 stability. As regulation of neuron growth by glutamate is concentration dependent^39^, we and others showed that tumor cells proliferation is stimulated by glutamate and limited by glutamate antagonists^38^. These results implicated the important role of acetylation of GluR1/2 in cell proliferation.

We further investigated the mechanism underlying acetylation-dependent glutamate-induced cell proliferation. Firstly, we observed that glutamate treatment of cells induced increased protein level of p70/p85 S6 kinase, which is required for accelerated protein synthesis in rapid cell growth and proliferation^40,41^, suggesting the involvement of mTOR pathway in this process, and consistent with previous report showing activation of mTOR by glutamate^42–45^. Next, we found that acetylation of GluR1/2 enhanced recruitment of β-arrestin1/2 and STAT3 to form a protein signalosome, from which glutamate-induced signaling bifurcated. β-Arrestin1/2 have been shown to transduce activation signal to mTOR and ERK1/2, respectively^46,47^. β-Arrestin2 mediates metabotropic GluR5-stimulated protein synthesis and downregulation of β-arrestin2 disrupts mGluR5-mediated ERK1/2 activation in the hippocampus^48^. In nonneuronal cells, β-arrestin1/2 also stimulates activation and sequestration of ERK1/2 in the cytosol^49,50^. β-Arrestin1 interacts with and activates STAT3 and is required for Th17 cell differentiation^28^. However, how the role of acetylation is implicated in the signal transduction function of β-arrestin1/2 is not clear. We therefore moved forward to show acetylation of β-arrestin1/2 enhanced signalosome formation and downstream activation of mTOR, ERK1/2, and STAT3. Put together our discoveries with other’s reports, acetylation of β-arrestin1/2 strengthened extracellular signaling mediated by GluR1/2.

Notably, we showed the upregulation of STAT3 acetylation at K685 and simultaneous translocation of acetylated STAT3 into mitochondria upon glutamate stimulation. Acetylation at K685 has been shown to be required for STAT3 translocation into mitochondria under stressed conditions to adjust pyruvate metabolism and ATP synthesis^17^. Therefore, upon glutamate stimulation, the purpose of STAT3 acetylation and translocation into mitochondria probably is to improve energy metabolism for cell growth and proliferation. Furthermore, glutamate also induced phosphorylation of STAT3 at Y705, and activated STAT3 can activate mTOR and vice versa^51,52^. Thus, our data provided a possibility that cross talk between STAT3 and mTOR is accurately regulated by GluR1/2-mediated signal transduction.

Our findings presented here revealed a novel signaling pathway that acetylation-dependent coordination of signalosome formation, including GluR1/2, β-arrestin1/2, and STAT3 is crucial to reinforce glutamate-induced cell growth and proliferation.

## Materials and methods

### Cell culture and reagents

C6, HeLa, SH-SY5Y, 239 T, and normal fibroblasts cells were obtained from the American Type Culture Collection and grown according to American Type Culture Collection recommendations. The entire above cell lines, as well as STAT3^−/−^ and control MEFs (obtained from X.Y. Fu) were cultured in 90% DMEM, 10% FBS (10099-141, Gibco), 100 μg/mL penicillin (15140-155, Life Technologies), and 100 μg/mL streptomycin (15140-122, Life Technologies) at 37 °C and 5% CO_2_.

### Plasmids

The plasmids CBP, P300, GluR1-Flag, GluR2-Flag, GluR2-HA, GluR2-Myc, Myc-MyD88, Loxl2-Flag, PICK1-Flag, STAT3-Myc, Arres1-Myc, and Arres2-Myc were granted by Dr. Chin’s lab in SJTU. GluR2-GFP, STAT3-RFP, all the different truncated mutants, and point mutations of GluR1 and GluR2 were constructed in our laboratory.

### Chemicals

DAPI (5D8417), glutamate (49621), and AMPK (SRP5003) were obtained from Sigma-Aldrich. Mito-Tracker Green (C1048) was obtained from Beyotime Biotechnology (Shanghai). PhTx-74 (ab120257) was obtained from Abcam.

### Antibodies

GluR1 antibody (SC-55509), GluR2 antibody (SC-517265), STAT3 antibody (SC-482), pY705-STAT3 antibody (SC-7993), c-Myc antibody (SC-40), and HA-probe antibody (SC-7392) were from Santa Cruz Biotechnology (Santa Cruz). Acetylated-lysine antibody (9441), acK685-STAT3 antibody (2523), pS727-STAT3 antibody (9136), P70/85 S6 kinase antibody (9202), pERK1/2 antibody (9106), and ERK1/2 antibody (9102) were from Cell Signaling Technology (Boston). H3 antibody (ab1791) and VDAC1/porin antibody (ab14734) were from Abcam. Flag antibody (F9291) and tubulin antibody (T2200) were from Sigma.

### Preparation of cytoplasmic, nuclear, and mitochondrial protein extracts

Cytoplasmic, nuclear, and mitochondrial extracts fractions were prepared by following the manufacturer’s instruction of cytoplasmic, nuclear extraction kit (P0027), and mitochondrial extraction kit (C3601), both bought from Beyotime technology. Briefly, 5 × 10^6^ cells were routinely used for grinding with 2 mL glass homogenate in ice bath. Cytoplasmic, mitochondrial, and nuclear fractions were separated through differential centrifugations. The supernatant and pellet were collected after the first centrifugation (600 × *g*, 10 min, 4 °C). The previous supernatant was centrifuged (10,000 × *g*, 10 min, 4 °C) to yield the supernatant (cytosolic fraction) and pellet (mitochondrial fraction). The first pellet was further lysed to yield the final nuclear lysate after centrifugation (600 × *g*, 10 min, 4 °C). To confirm that purity of extracts was obtained, mitochondria, nuclear, and cytoplasmic fractions were separated by SDS–PAGE and the presence of the mitochondria protein porin, nuclear protein H3, or the cytoplasmic protein tubulin was demonstrated by western blot analysis, using monoclonal mouse antibodies.

### Western blotting and co-immunoprecipitation

About 2 × 10^6^ cells after disposed by different conditions were lysed in 150 μL western and IP lysate buffer (P0013, Beyotime) containing 50 mM Tris (pH 7.4), 150 mM NaCl, 1% Triton X-100, 1% NP-40, and multiple inhibitors (phosphatase inhibitors, sodium pyrophosphate, sodium fluoride, EDTA, leupeptin, etc). A total of 20 μL protein lysate were separated for western blotting. The rest part was incubated with 1.0 μg required antibody and 20 μL protein A/G PLUS-Agarose beads (SC-2003, Santa Cruz) at 4 °C for overnight. Proteins were fractionated by SDS–PAGE and transferred to a nitrocellulose membrane. Membranes were blocked with 5% BSA in Tris-buffered saline and incubated with the primary antibodies at 4 °C for overnight. Blots were developed with a peroxidase-conjugated fluorescence secondary antibody for 1 h at room temperature, and then the western blotting results were scanned and analyzed by LI-COR system (Odyssey).

### RNA-seq

About 1 × 10^7^ C6 cells were treated with or without glutamate. The total protein of every cell sample was extracted for the further RNA-seq analysis. RNA-seq analysis was carried out by Yunxu Biotechnology Co., Ltd (Shanghai).

### Mass spectrometry analysis

About 2 × 10^7^ 293 T cells had been transfected by GluR1 or GluR2 for 48 h were splited by RIPA lysate solution (P0013, Beyotime). Trypsin solution (T6567, Sigma) was added to the protein sample in total volume of 100 μL and incubated at 37 °C overnight. Trypsin lysates were added to Amicon Ultra-0.5 mL Centrifugal Filter Unit (Ultracel-10K), centrifuged at 13,000 r.p.m., 4 °C, 10 min, dried the pellets with Vacuum freeze dryer (Millipore). Resuspended samples in buffer A (Ultra-Pure water with 0.1% formic acid), measured peptide concentration, and proceeded to LC-Ms/Ms analysis (6410, Agilent).

### Immunofluorescence staining

Cells which had been inoculated on glass coverslips were respectively transfected by GluR2-GFP or STAT3-RFP for 36 h. Cells with different treatment were fixed and permeabilized in ice-cold paraformaldehyde and then blocked with phosphate-buffered saline-1% bovine serum albumin at room temperature. Figure 4e, STAT3 was localized using STAT3 antibody (C-20, Santa Cruz) at 4 °C overnight, followed by incubation with goat anti-rabbit immunoglobulin G-Alexa Fluor 488 secondary antibody (A0423, Beyotime) at room temperature for 2 h. At last, stained cells with DAPI staining solution for 5 mins, observed and analyzed cells using inverted fluorescence microscope (OLYMPUS).

### Cell proliferation

STAT3 WT MEF, STAT3^−/−^ MEF, C6, and HeLa cells seeded in 12 wells cell culture plate were treated by glutamate. Cells were observed and analyzed by inverted fluorescence microscope (OLYMPUS).

### Statistical analysis

Continuous values were presented as SDs (means and standard deviations). Statistical analyses were performed through Windows office version 2007 Excel statistical software. IBM SPSS statistical software version 22 ensured *p < 0.05, and as highly statistically significant if **p < 0.01 to indicate statistical significance.
